# Supplementary material for: BcSOC1 Promotes Bolting and Stem Elongation in Flowering Chinese Cabbage
Source: Int J Mol Sci. 2022 Mar 22;23(7):3459. doi: 10.3390/ijms23073459 (PMC8998877; doi:10.3390/ijms23073459)
Supplement: Supplementary file 1 [file ijms-23-03459-s001.zip › ijms-1629124-supplementary/Supplementary_material/Supplementary_material.pdf]

Supplementary Figures

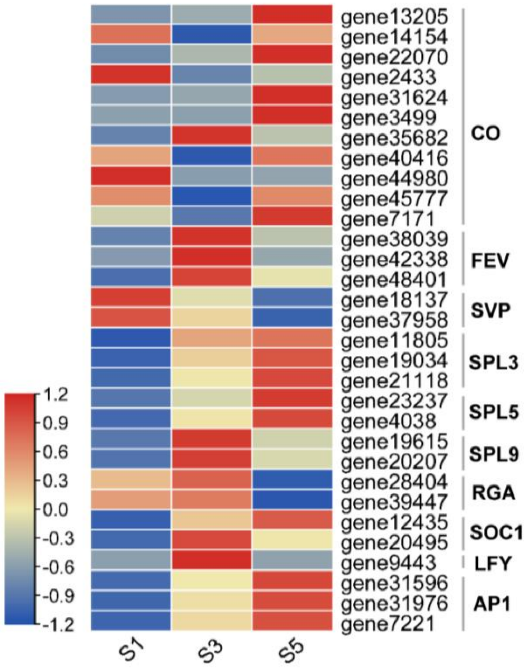

Figure S1. Heat-map of flowering-related genes that were differentially expressed during the three stalk developmental stages (S1, S3, and S5) (Huang et al., 2017). Red and blue colors represent up- and downregulation, respectively. Values represent z-scores of the FPKM data.

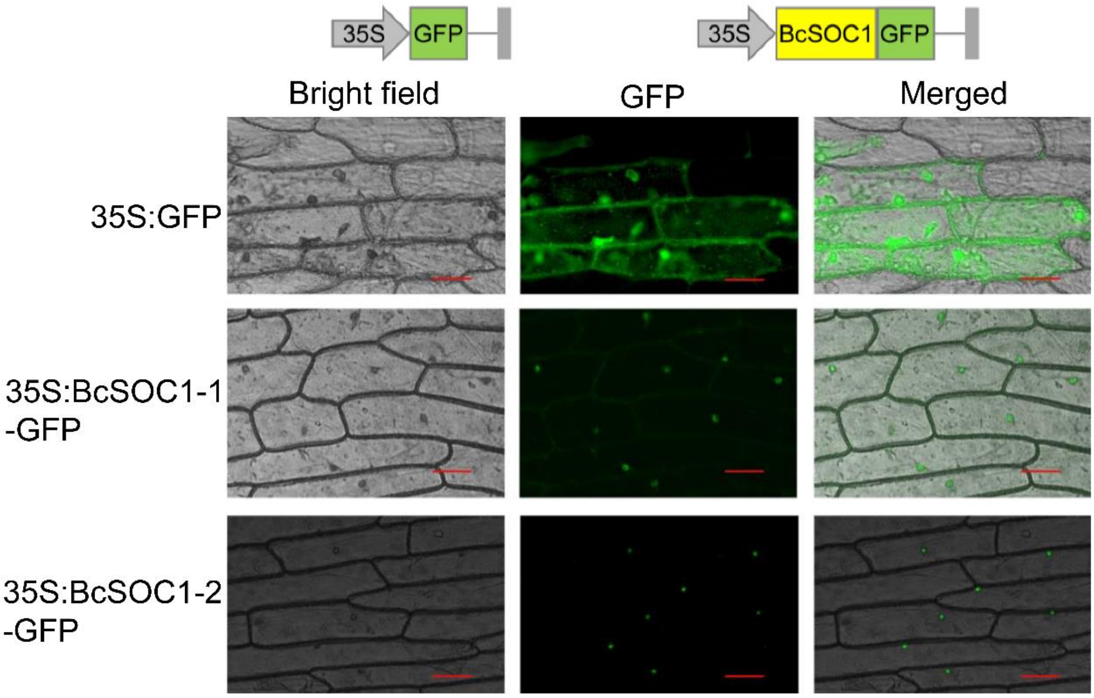

Figure S2. Subcellular localization of *BcSOC1* in onion epidermal cells. Bar = 100 μm.

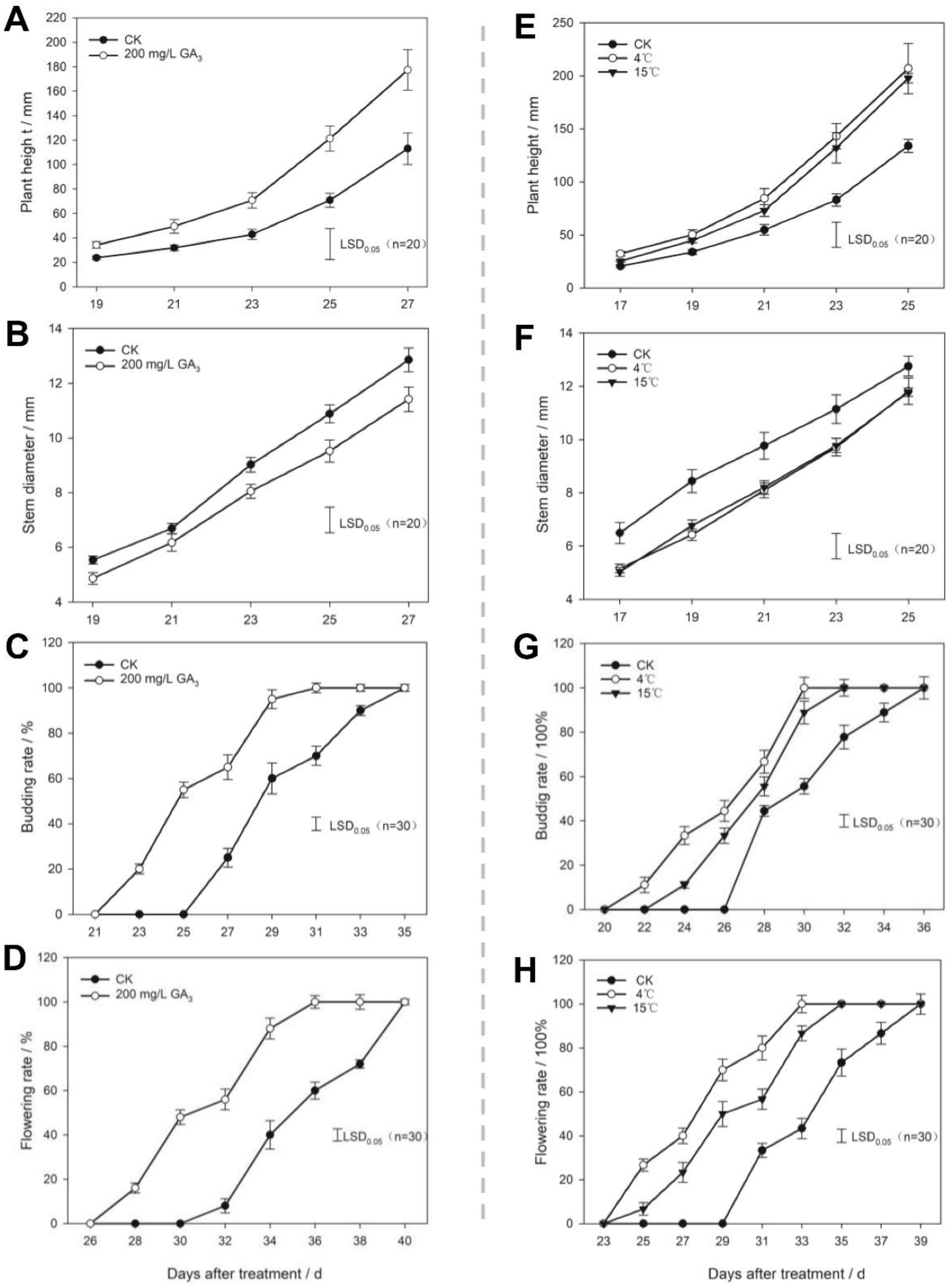

Figure S3. Effects of GA_3 and low-temperature treatments on the growth stages and bolting in flowering Chinese cabbage. **(A–D)** Plant height, stem diameter, budding rate, and flowering rate of flowering Chinese cabbage treated with GA_3. **(E–F)** Plant height, stem diameter, budding rate, and flowering rate of flowering Chinese cabbage exposed to low temperatures (4 ºC and 15 ºC). CK as the control. Different letters indicate significant differences ($p < 0.05$) determined using ANOVA.

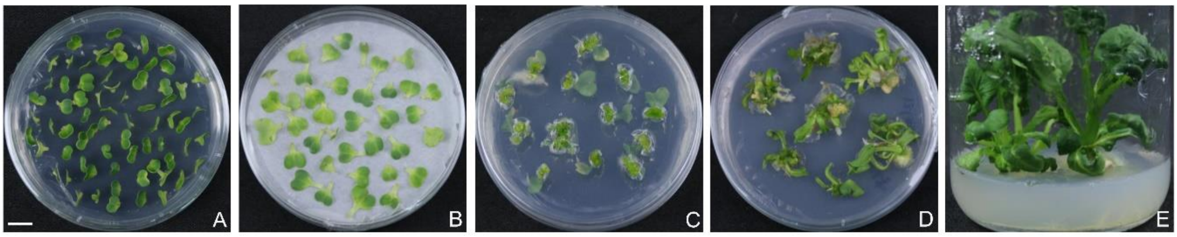

Figure S4. *Agrobacterium*-mediated transformation in flowering Chinese cabbage. (**A**) Pre-culture; (**B**) co-culture; (**C**) delayed screening; (**D**) resistance screening; (**E**) generated roots. Bar = 1 cm.

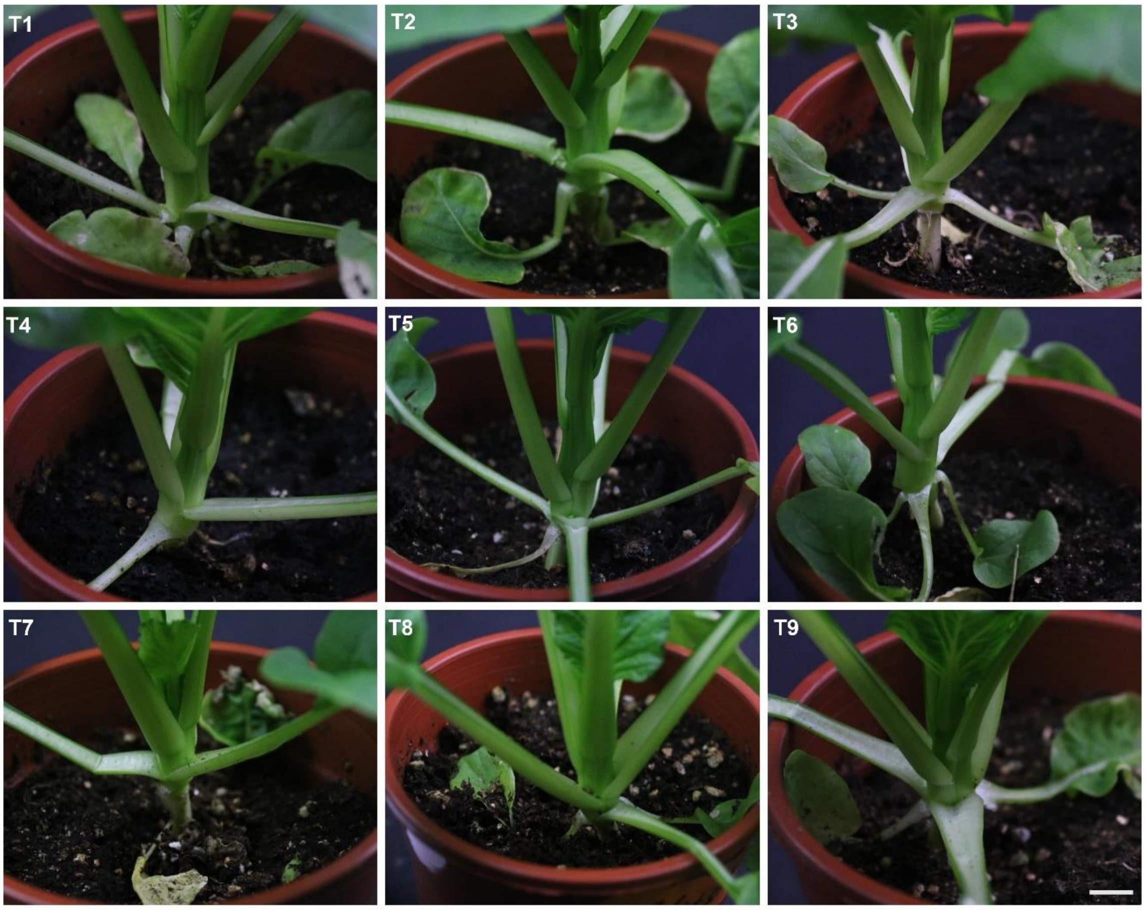

Figure S5. Stalk morphology of *BcSOC1* knockdown lines shown in Figure 4. Bar = 1 cm.

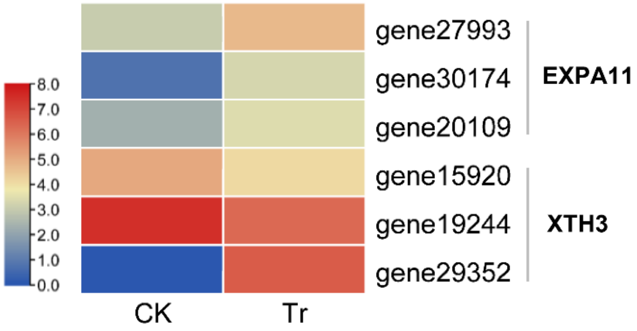

Figure S6. Heat map of *BcEXPA11* and *BcXTH3* in the control (CK) and low-temperature (Tr; ºC) treatments. Red and blue colors represent up- and down-regulation, respectively.

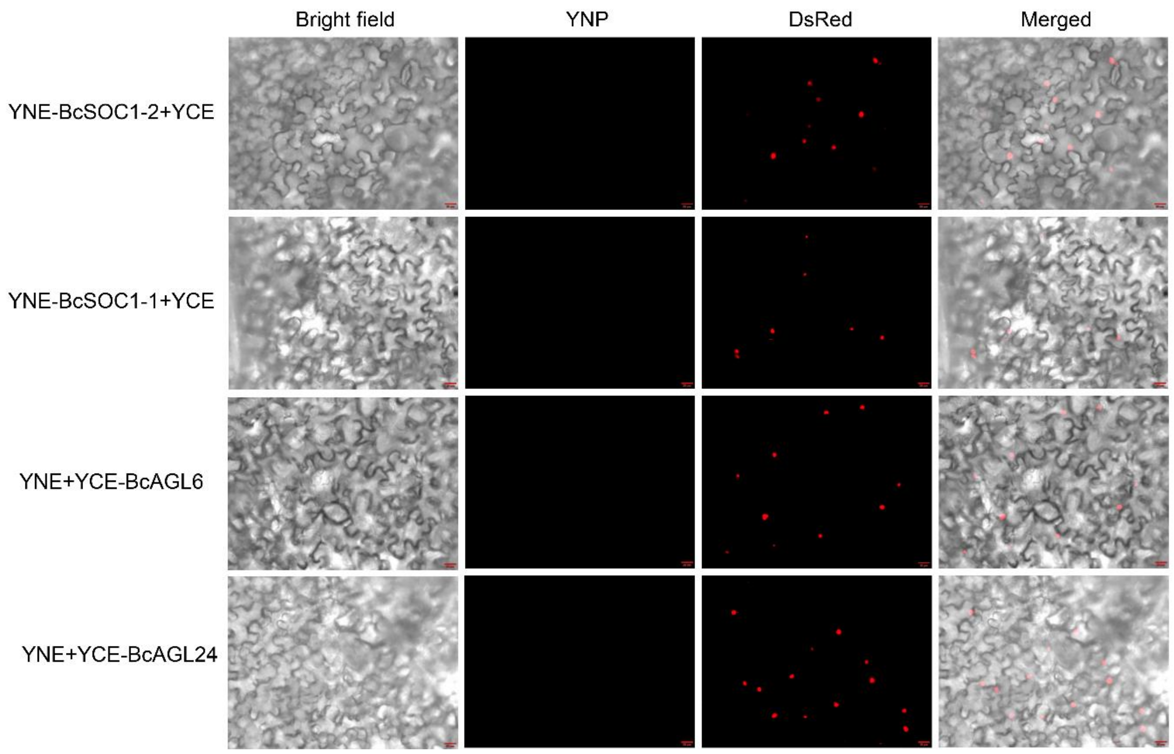

Figure S7. Expression levels of BcSOC1-1, BcSOC1-2, BcAGL6, or BcAGL24 were used as negative controls in the bimolecular fluorescence complementation assay. Bar = 50 μm.

Supplementary Tables

Table S1. List of the primer for cloning

	**Primer name**	**Sequence**
BcSOC1-1	BcSOC1-1-F	ATGGTGAGGGGCAAAACTCA
	BcSOC1-1-R	TCACTTTCTTGAAGAACAAGGTAACC
BcSOC1-2	BcSOC1-2-F	ATGGTGAGGGGCAAAACTCA
	BcSOC1-2-R	TCACTTTCTTGAAGAACAAGGTAACC

Table S2. List of the primer for qRT-PCR

	Primer name	Sequence
BcSOC1-1	BcSOC1-1-RT-F	GAAGGCATTGGATCATGCTCGATTG
	BcSOC1-1-RT-R	TACTGTCCTCGTCACCTCGTCCACT
BcSOC1-2	BcSOC1-2-RT-F	GAAGGCATAGGATCATGTTCGATAG
	BcSOC1-2-RT-R	TACTCTCTTCGTCACCTTTTCCACT
BcLFY	BcAGL6-RT-F	CGAACAGTGTCGCGAGTTTC
	BcAGL6-RT-R	GTAGTGTCGCATCTTCGGCT
BcEXPA11	BcEXPA11-RT-F	GAACTTCGCTCTCCCCAACA
	BcEXPA11-RT-R	TTCCGATCTTCTCCCAAGCG
BcXTH3	BcXTH3-RT-F	CCAGTCTCTGGAACGGTGAC
	BcXTH3-RT-R	CTGACAGGCCGGAAAAGTCT

Table S3. List of the primer for subcellular localization

	Primer name	Sequence
BcSOC1-1	BcSOC1-1-GFP-F	ctgcccaaattcgcgaccggtATGGTGAGGGGCAAAACTCA
	BcSOC1-1-GFP-R	gcccttgctcaccataccggtCTTTCTTGAAGAACAAGGTAACCCA
BcSOC1-2	BcSOC1-2-GFP -F	ctgcccaaattcgcgaccggtATGGTGAGGGGCAAAACTCA
	BcSOC1-2-GFP-R	gcccttgctcaccataccggtCTTTCTTGAAGAACAAGGTAACCCA

Table S4. List of the primer for construction of overexpression and VIGS vectors

	Primer name	Sequence
BcSOC1-1	BcSOC1-1-OE-F	gagaacacgggggactctagaATGGTGAGGGGCAAAACTCA
	BcSOC1-1-OE-R	ggactgaccacccggggatccCTTTCTTGAAGAACAAGGTAACC
	BcSOC1-1-VIGS-F	aaggttaccgaattctctagaGGCATTGGATCATGCTCGATTG
	BcSOC1-1-VIGS-R	tgtcttcgggacatgcccgggTCACTTTCTTGAAGAACAAGGT
BcSOC1-2	BcSOC1-2-OE-F	gagaacacgggggactctagaATGGTGAGGGGCAAAACTCA
	BcSOC1-2-OE-R	ggactgaccacccggggatccCTTTCTTGAAGAACAAGGTAACC
	BcSOC1-2-VIGS-F	aaggttaccgaattctctagaGACTTTCTCTAAGCGAAGGAAT
	BcSOC1-2-VIGS-R	tgtcttcgggacatgcccgggCATGATCCTATGCCTTCTCCCA

Table S5. List of the primer for yeast two-hybrid assays

	**Primer name**	**Sequence**
BcSOC1-1	AD-BcSOC1-1-F	gccatggaggccagtgaattcATGGTGAGGGGCAAAACTCA
	AD-BcSOC1-1-R	cagctcgagctcgatggatccTCACTTTCTTGAAGAACAAGGTAACC
	BD-BcSOC1-1-F	atggccatggaggccgaattcATGGTGAGGGGCAAAACTCA
	BD-BcSOC1-1-R	ccgctgcaggtcgacggatccTCACTTTCTTGAAGAACAAGGTAACC
BcSOC1-2	AD-BcSOC1-2-F	gccatggaggccagtgaattcATGGTGAGGGGCAAAACTCA
	AD-BcSOC1-2-R	cagctcgagctcgatggatccTCACTTTCTTGAAGAACAAGGTAACC
	BD-BcSOC1-2-F	atggccatggaggccgaattcATGGTGAGGGGCAAAACTCA
	BD-BcSOC1-2-R	ccgctgcaggtcgacggatccTCACTTTCTTGAAGAACAAGGTAACC
BcAGL6	AD-BcAGL6-F	gccatggaggccagtgaattcATGGGAAGAGGGAGAGTGGAGA
	AD-BcAGL6-R	cagctcgagctcgatggatccTCAAAGAACCCAACCTTGGACG
	BD-BcAGL6-F	atggccatggaggccgaattcATGGGAAGAGGGAGAGTGGAGA
	BD-BcAGL6-R	ccgctgcaggtcgacggatccTCAAAGAACCCAACCTTGGACG
BcAGL24	AD-BcAGL24-F	gccatggaggccagtgaattcATGGCGAGAGAGAAGATAAGGATAA
	AD-BcAGL24-R	cagctcgagctcgatggatccTCATTCCCAAGATGGAAGCCC
	BD-BcAGL24-F	atggccatggaggccgaattcATGGCGAGAGAGAAGATAAGGATAA
	BD-BcAGL24-R	ccgctgcaggtcgacggatccTCATTCCCAAGATGGAAGCCC

Table S6. List of the primer for BIFC assays

	Primer name	Sequence
BcSOC1-1	YNE-BcSOC1-1-F	cgcgccactagtggatccATGGTGAGGGGCAAAACTCA
	YNE-BcSOC1-1-R	ggtaccctcgaggtcgacCTTTCTTGAAGAACAAGGTAACC
BcSOC1-2	YNE-BcSOC1-2-F	cgcgccactagtggatccATGGTGAGGGGCAAAACTCA
	YNE-BcSOC1-2-R	ggtaccctcgaggtcgacCTTTCTTGAAGAACAAGGTAACC
BcAGL6	YCE-BcAGL6-F	cgcgccactagtggatccATGGGAAGAGGGAGAGTGGAGA
	YCE-BcAGL6-R	ggtaccctcgaggtcgacAAGAACCCAACCTTGGACG
BcAGL24	YCE-BcAGL24-F	cgcgccactagtggatccATGGCGAGAGAGAAGATAAGGATA A
	YCE-BcAGL24-R	ggtaccctcgaggtcgacTTCCCAAGATGGAAGCCC
